# A synopsis of Estonian myriapod fauna (Myriapoda: Chilopoda, Diplopoda, Symphyla and Pauropoda)

**DOI:** 10.3897/zookeys.793.28050

**Published:** 2018-10-29

**Authors:** Kaarel Sammet, Mari Ivask, Olavi Kurina

**Affiliations:** 1 Institute of Agricultural and Environmental Sciences, Estonian University of Life Sciences, Kreutzwaldi st 5D, 51006 Tartu, Estonia Estonian University of Life Sciences Tartu Estonia; 2 Tallinn University of Technology, Tartu College, Puiestee 78, 51008 Tartu, Estonia Tallinn University of Technology Tartu Estonia

**Keywords:** check list, Chilopoda, Diplopoda, distribution, Estonia, Myriapoda, Pauropoda, soil invertebrates, Symphyla

## Abstract

The data on Estonian Myriapoda are scattered in various publications and there has been no overview of the fauna up to the present. A critical summary of the previous information on Estonian Myriapoda is given, supplemented by new records and distribution maps. Altogether, 5784 specimens from 276 collecting sites were studied. To the hitherto recorded 14 centipede species are added *Lithobiusmelanops*, *L.microps*, *Geophiluscarpophagus*, *G.flavus*, *Strigamiatranssilvanica* and *Stenotaenialinearis*, a probably introduced species. Of the 27 published Estonian millipede species, the data on two species proved erroneous, and two new species were recorded (*Craspedosomaraulinsii* and *Cylindroiulusbritannicus*). Two previously recorded millipede species – *Brachyiuluspusillus* and *Mastigophorophyllonsaxonicum* – were not found in the recent samples, the latter may have become more rare or extinct. Pauropoda and Symphyla lack previous reliable records. Combined with published data, the number of myriapod species known from Estonia is now set at 52. Some changes in species distribution and frequencies were detected comparing the published data with new records. Some data about habitat preferences of the more common species are also given. The majority of species have a western Palaearctic distribution, while six species are at the northern limit of their ranges.

## Introduction

The research of Estonian Myriapoda has been quite unsystematic and sporadic. Very little has been published in English thus much of the information may be currently unavailable to the wider myriapodological community (e.g., Zapparoli 2003, Tuf et al. 2015). The first scant records of Myriapoda in Estonia date back to the second half of the XIX century. The first data are given by E. Haase (Haase 1886: 58), who mentions a “Craspedosomamutabilev.fasciatum Latzel, 1884” specimen collected by A. E. Grube from Tartu. Subsequently, P. Schmidt reported the presence of *PauropusHuxleyi* in the vicinity of Narva (Schmidt 1894) and O. Schubart published the data on two millipede species collected from Estonian bogs (Schubart 1924). W. Mierzeyewski collected in 1912 and 1926 some millipedes in the island Saaremaa, and in the years 1925 to 1929 W. Herold gathered a considerable millipede material from many places in Estonia. That material, containing 20 species, was also identified and published by O. Schubart (Schubart 1930), who repeated the data in his monograph on German Diplopoda (Schubart 1934). Part of the Herold material is currently preserved in the collection of Museum für Naturkunde, Berlin. In the 1930’s, some ecological studies mention millipedes identified to genus level (e.g., Nõmmik 1939) and some species were listed in studies on plant pests (e.g., Zolk 1923, Kaarep et al. 1949). E. Palmén published one new record of Estonian diplopods in his overview of the Finnish fauna (Palmén 1949).

An unpublished collection of myriapods from 1937, preserved currently in the entomological collection of Estonian University of Life Sciences, Tartu (IZBE, identified by the Swedish zoologist H. Lohmander), has probably served as a basis to the list of ten centipede species in H. Riikoja’s account of Estonian invertebrates (Riikoja 1955; referred to as pers. comm. with J. Vilbaste).

We owe thanks for much of what is known about Estonian Myriapoda to the works of the Estonian entomologist Juhan Vilbaste (1924–1985). His “Keys to Estonian Millipedes” lists 21 species as proven to occur in Estonia at that time (Vilbaste 1953). In addition, he published on Myriapoda in several local faunistic surveys, adding one centipede and five millipede species records and some ecological observations (Vilbaste 1970, Vilbaste 1979, Vilbaste et al. 1985, Vilbaste and Vilbaste 1993). Unfortunately, only two specimens of Vilbaste have subsisted (in the IZBE collection). The data provided by Schubart (1930) and Vilbaste (1953) have been reproduced by various subsequent authors (e.g., Lang 1954, Stojałowska 1961, Lokshina 1969). Thus, 14 centipede species and 27 millipede species were recorded from Estonia prior to the current study.

## Material and methods

As complete as possible, bibliography of historical records of myriapods in Estonia was compiled, reviewing all the available faunistic studies and other records. The main Estonian zoological collections were searched for myriapod material (Estonian Museum of Natural History, Tallinn; Tartu University Museum of Natural history and the private insect collection of Allan Selin, Maardu). Some collections abroad known to house Estonian material were contacted for further information (Finnish Museum of Natural History and Zoologische Staatssammlung München, Germany).

New material was collected using: (1) pitfall traps, (2) Tullgren funnel and Kempson apparatus, (3) sifting moss, leaf litter and detritus with a standard entomological sieve, (4) manual searching in suitable habitats and daytime retreats, and (5) as by-catch of non-target species with window pane traps (attached to tree trunks) and Malaise traps (for particular description of the trapping projects, see Sammet et al. 2016 and Tomasson et al. 2014, respectively).

**Figure 1.**
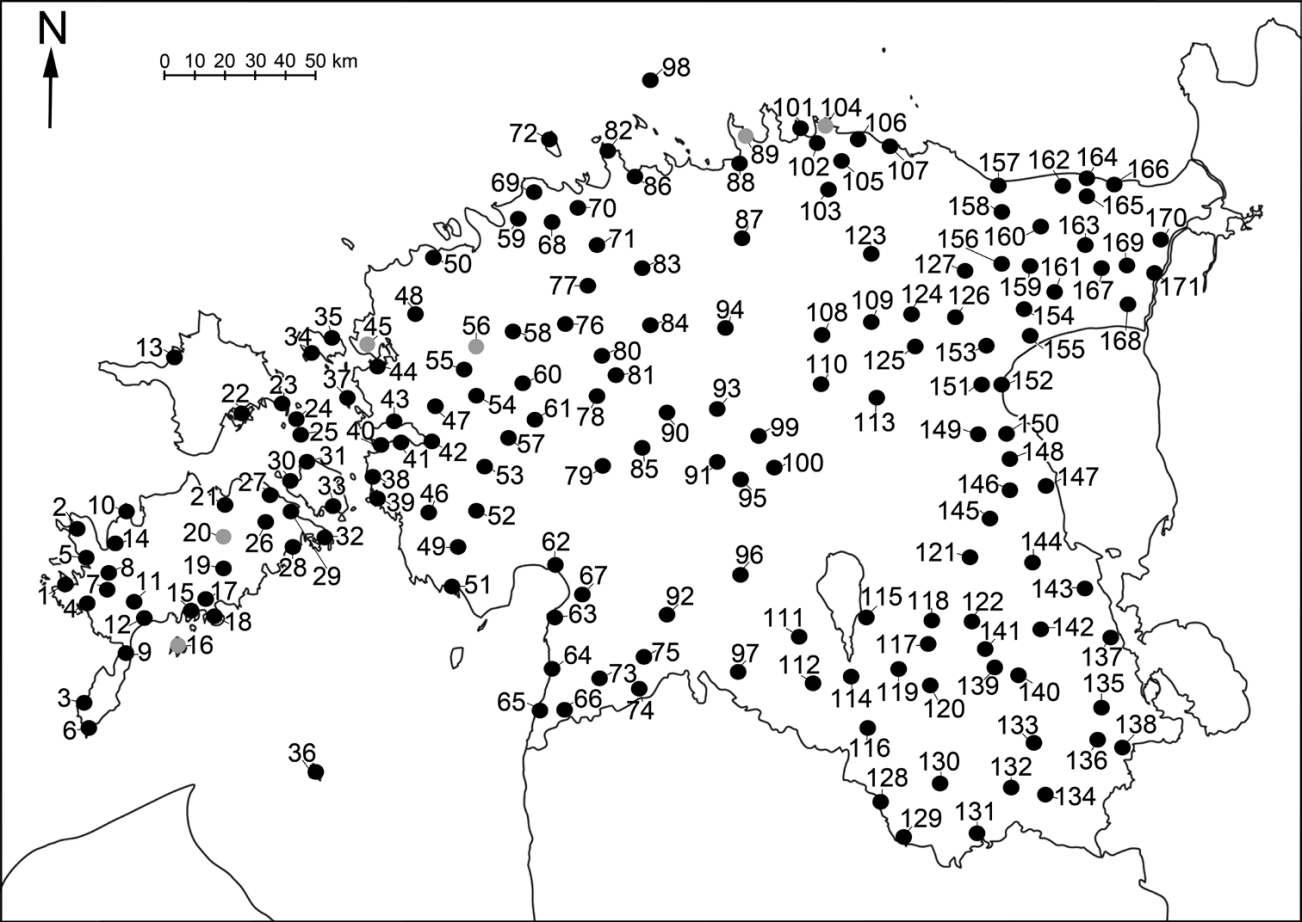
Collecting localities of Myriapoda in Estonia. Key: Dark circles = this study, light circles = literature data. For further details, see Table 1.

**Figure 2.**
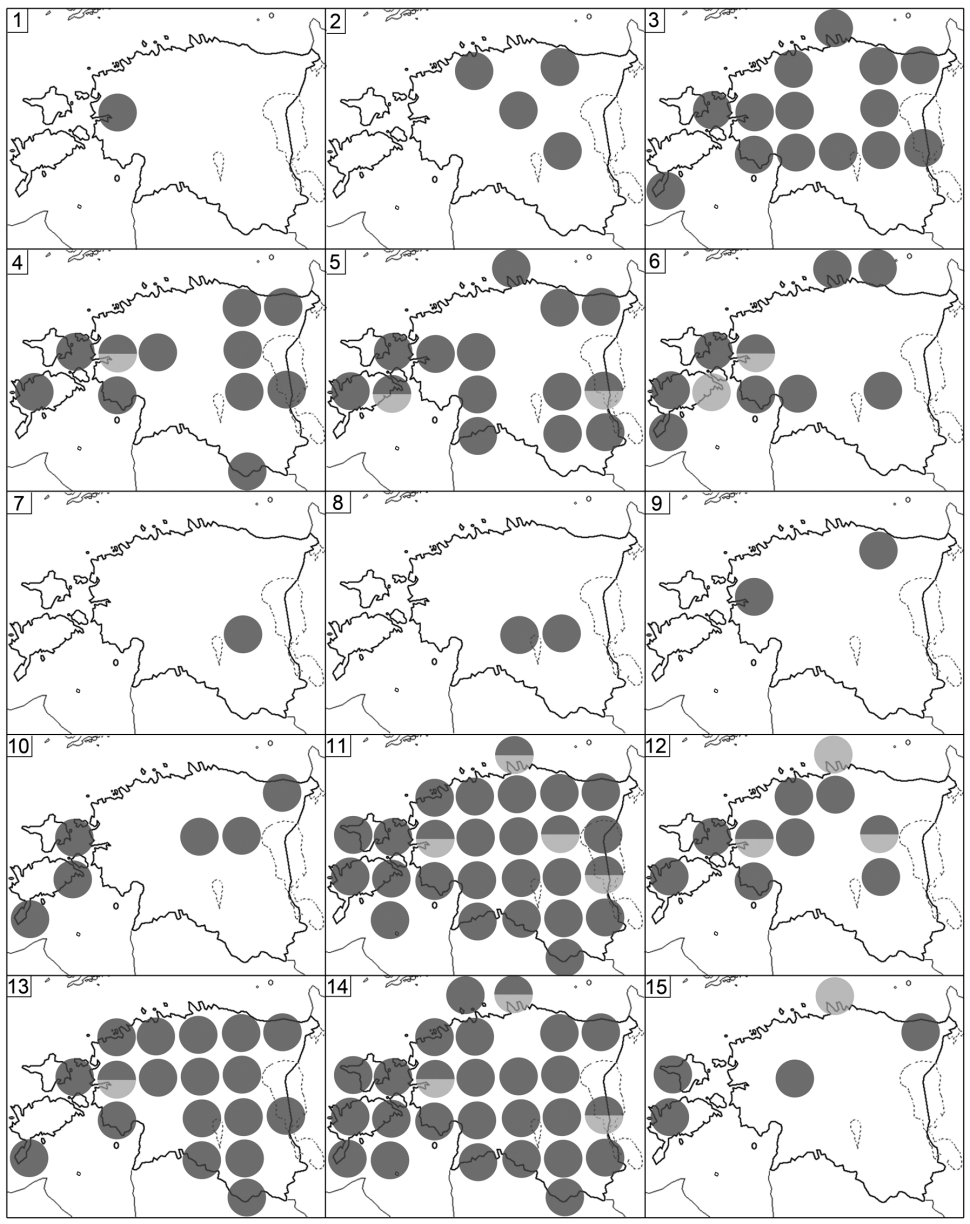
Distribution of Estonian Chilopoda. **1***Geophiluscarpophagus***2***G.electricus***3***G.flavus***4***G.proximus***5***G.truncorum***6***Pachymeriumferrugineum***7***Stenotaenialinearis***8***Strigamiatranssilvanica***9***Lamyctesemarginatus***10***Lithobiusborealis***11***L.curtipes***12***L.crassipes***13***L.erythrocephalus***14***L.forficatus***15***L.lucifugus*. Key: Dark circles = original data, light circles = literature data, divided circles = original and literature data.

**Figure 3.**
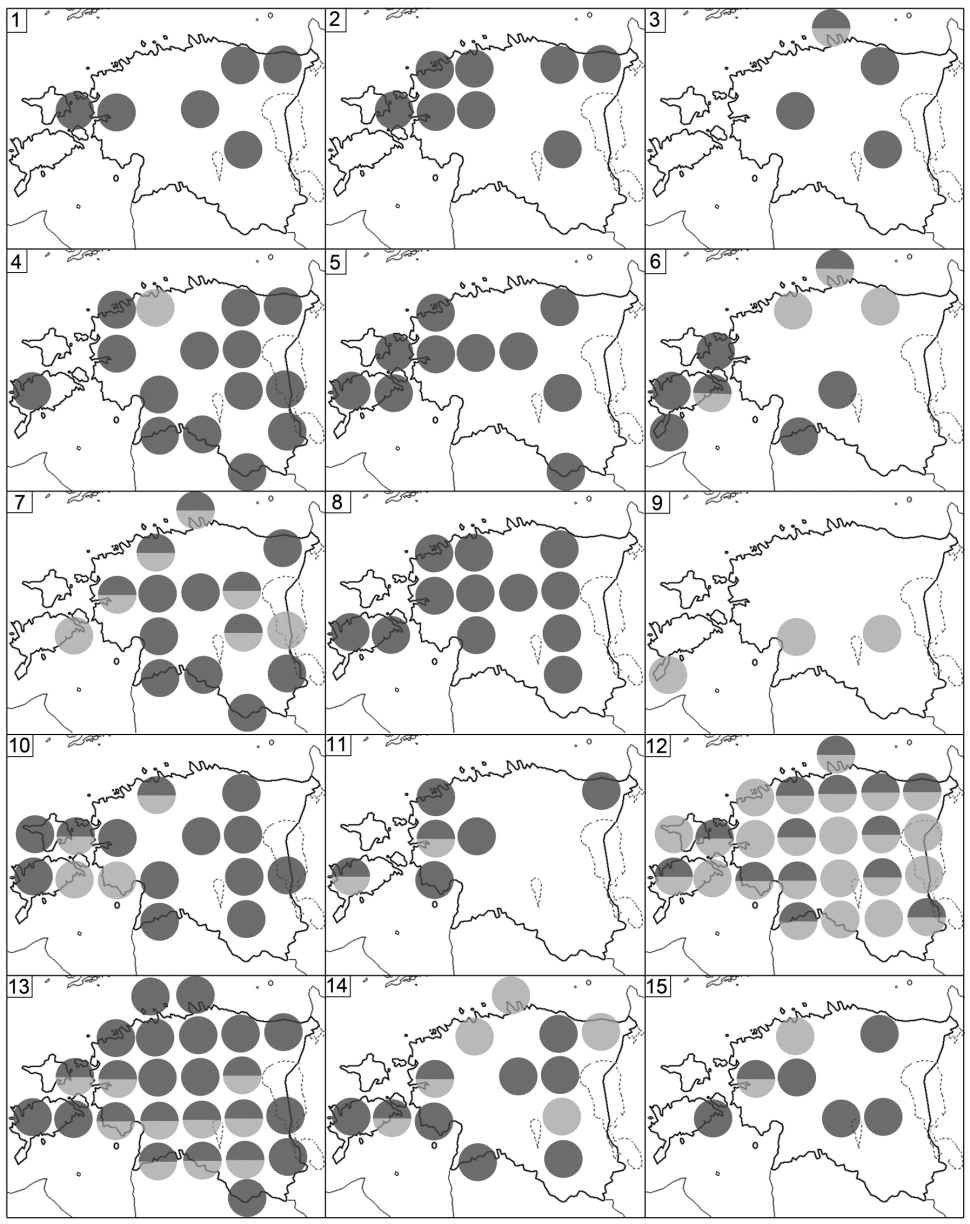
Distribution of Estonian Chilopoda (**1–5**) and Diplopoda (**6–15**). **1***Lithobiusmelanops***2***L.microps***3***L.pelidnus***4***L.tenebrosus***5***Schendylanemorensis***6***Polyxenuslagurus***7***Polyzoniumgermanicum***8***Craspedosomaraulinsii***9***Mastigophorophyllonsaxonicum***10***Nemasomavaricorne***11***Brachydesmussuperus***12***Polydesmuscomplanatus***13***P.denticulatus***14***P.inconstans***15***Blaniulusguttulatus*. For symbols see Fig. 2.

**Figure 4.**
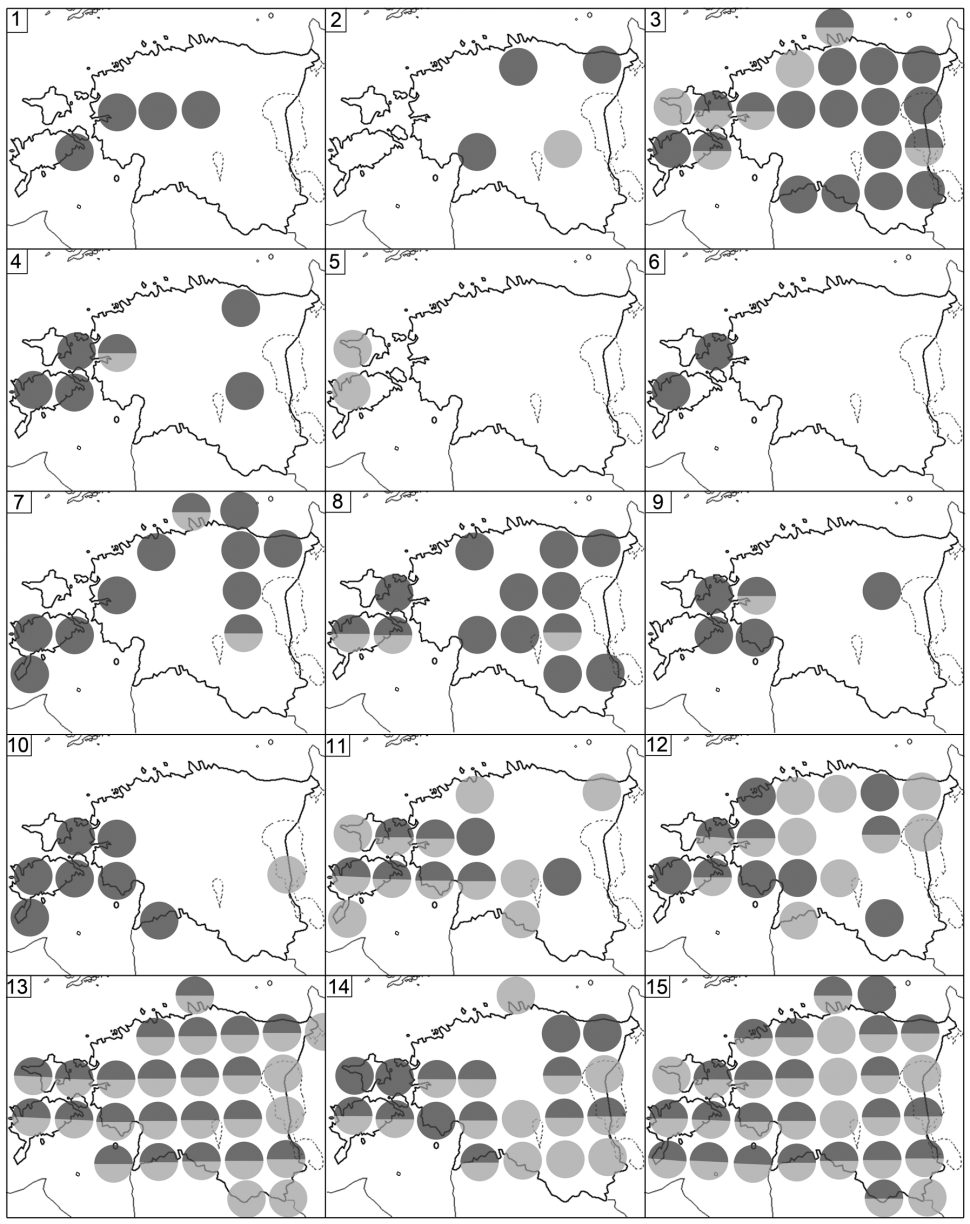
Distribution of Estonian Diplopoda. **1***Boreoiulustenuis***2***Nopoiuluskochii***3***Proteroiulusfuscus***4***Allajulusnitidus***5***Brachyiuluspusillus***6***Cylindroiulusbritannicus***7***C.latestriatus***8***C.caeruleocinctus***9***Julusscandinavius***10***J.scanicus***11***J.terrestris***12***Leptoiuluscibdellus***13***L.proximus***14***Megaphyllumsjaelandicum***15***Ommatoiulussabulosus*. For symbols see Fig. 2.

**Figure 5.**
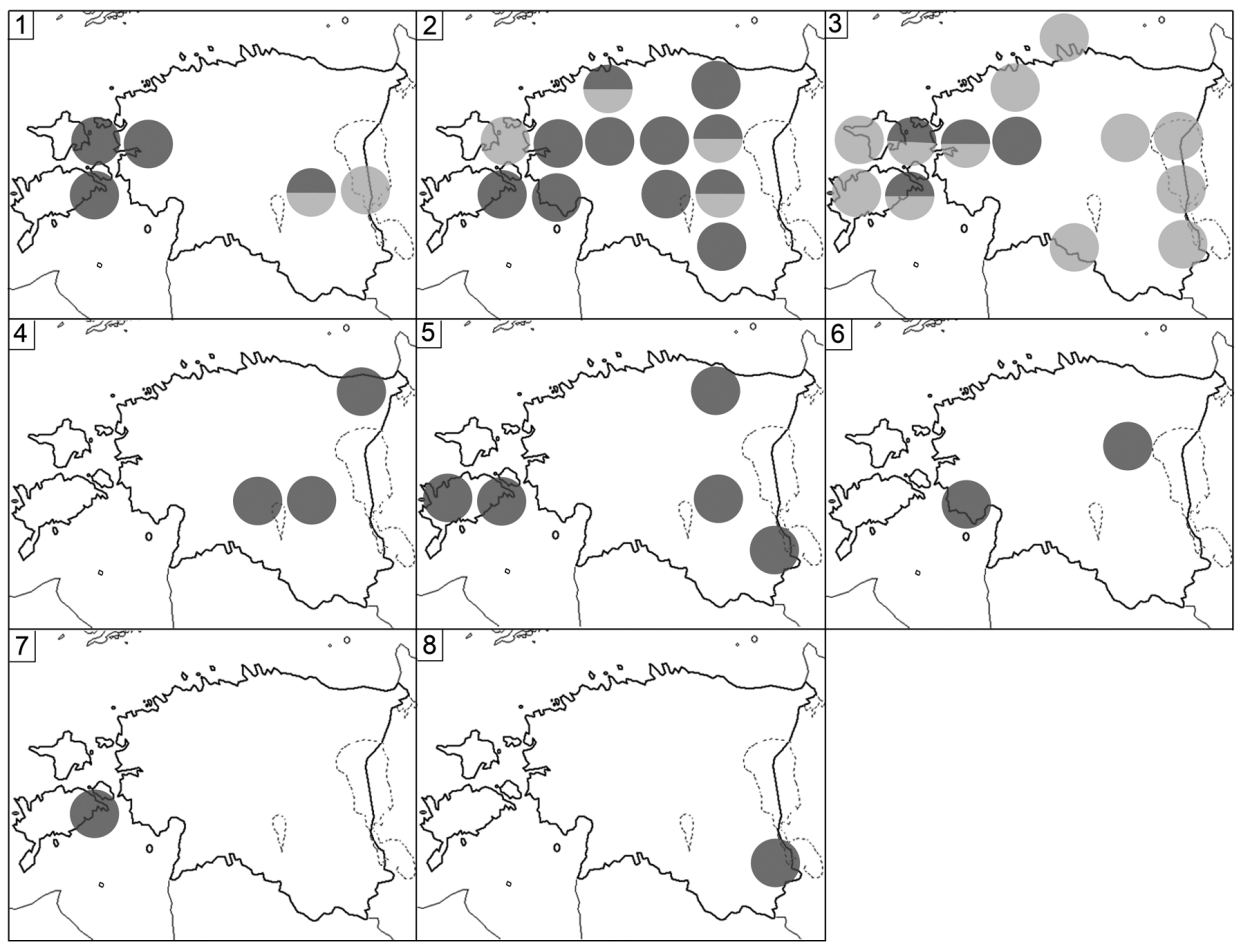
Distribution of Estonian Diplopoda (**1–3**), Symphyla (**4–6**) and Pauropoda (**7–8**). **1***Rossiulusvilnensis***2***Uncigerfoetidus***3***Xestoiuluslaeticollis***4***Scutigerellaimmaculata***5***Symphylellavulgaris***6***Scolopendrellopsissubnuda***7***Decapauropuscuenoti***8***D.gracilis*. For symbols see Fig. 2.

**Figure 6.**
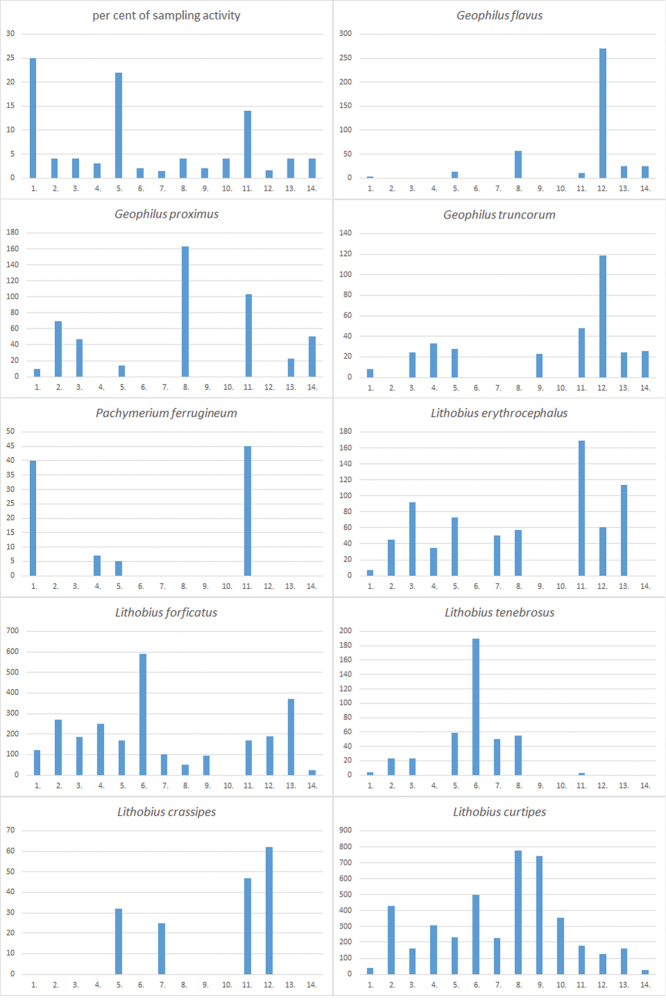
Proportion of samples from different habitats and habitat preferences of common Estonian Chilopoda. Vertical axis: relative abundances (numbers of findings divided by proportion of sampling effort). Horizontal axis numbers represent habitat types as follows: **1** Coastal meadows and alvars **2** Broad-leaved (nemoral) forests **3** Boreo-nemoral deciduous forests **4** Dry heathland forests **5** Drier boreo-nemoral mixed forests **6** Mesophilic boreal forests **7** Hillock forests **8** Carrs and swamp forests **9** Bogs **10** Fens and waterlogged meadows **11** Inland mesophilic grasslands **12** Rural gardens **13** Urban parks and graveyards **14** Arable fields. For detailed description of habitats see Material and methods.

**Figure 7.**
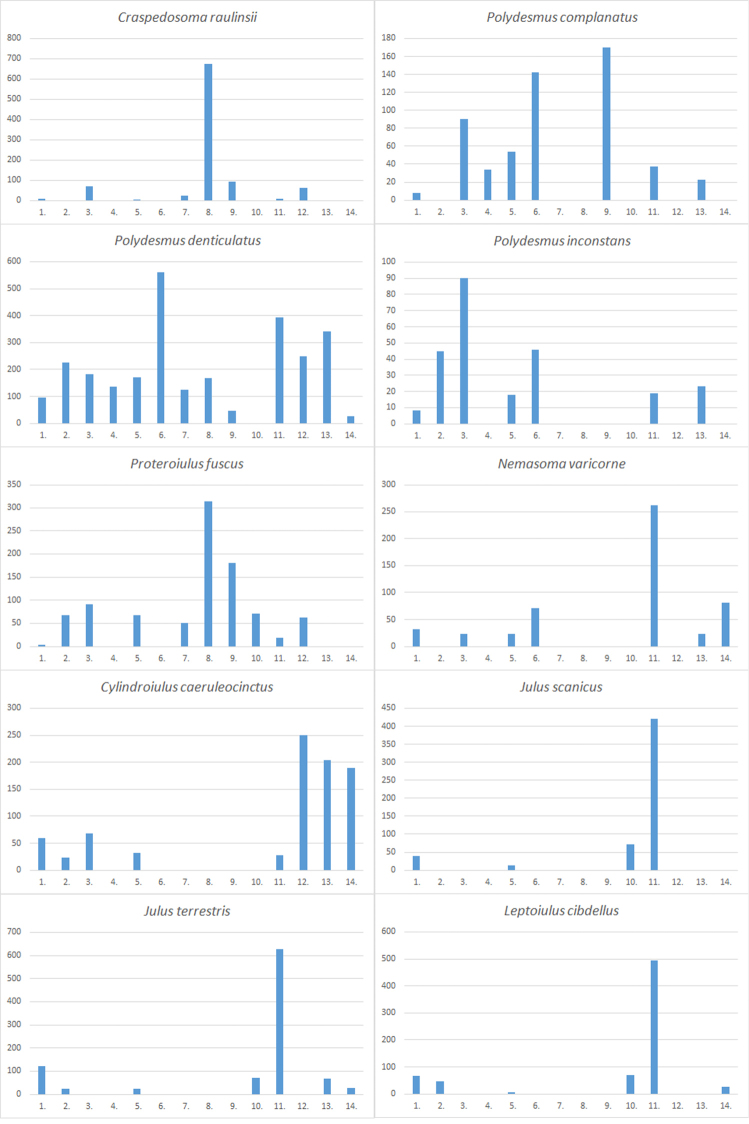
Habitat preferences of common Estonian Diplopoda. For explanation of the vertical axis and numbers denoting different habitats on the horizontal axis see Fig. 6.

**Figure 8.**
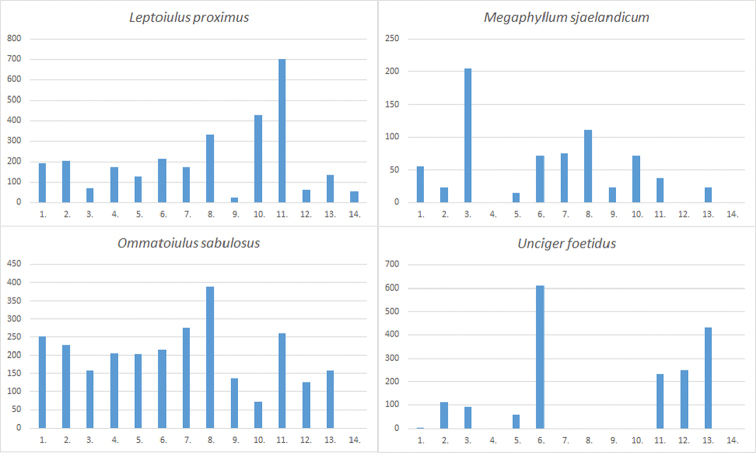
Habitat preferences of common Estonian Diplopoda. For explanation of the vertical axis and numbers denoting different habitats on the horizontal axis see Fig. 6.

The material was collected from 276 localities covering all parts of Estonia (see Table 1 and Figure 1 for details). The distribution of Estonian species (Figures 2–5) is presented in 50×50 km UTM grid, also used in the „Atlas of European Millipedes“ (Kime and Enghoff 2011, 2017) (compiled using Adobe Photoshop CS5 Extended). The relative abundances in different habitats of species with at least 25 findings were presented as diagrams (Figures 6–8). The habitats studied repeatedly with different methods were grouped into 14 types:

1. Coastal meadows and alvars;

2. Broad-leaved (nemoral) forests (dominated by *Quercusrobur*, *Tiliacordata*, *Acerplatanoides* and herbs in the understory);

3. Boreo-nemoral deciduous forests (dominated by *Alnusincana* or *Salix* species);

4. Dry heathland forests (dominated by *Pinussylvestris*, with *Cladonia* sp. or *Callunavulgaris* in understory);

5. Drier boreo-nemoral mixed forests (dominated by *Pinussylvestris* with *Sorbus* and *Acer* and *Vacciniummyrtillus* in understory);

6. Mesophilic boreal forests (dominated by *Piceaabies*, *Oxalisacetosella* in understory);

7. Hillock forests (dominated by *Corylusavellana*, herbs in the understory);

8. Carrs and paludifying forests (dominated by *Piceaabies*, *Betulapendula* and *Pinussylvestris*);

9. Bogs;

10. Fens and waterlogged meadows;

11. Inland mesophilic grasslands;

12. Rural gardens;

13. Urban parks and graveyards;

14. Arable fields.

The barplot diagrams were produced by dividing the number of findings in a habitat type by the proportion of sampling effort in that particular habitat (i.e. the number of “sampling events” consisting of one trapping period or one hand collecting trip with subsequent Tullgren extraction of soil and litter samples) (Figure 6). Other, more rare, habitats that were not studied with all the methods are not included. All studied material is preserved in 70% ethanol or a mix of ethanol and glycerol for Pauropoda. Some gonopods are preserved as microscope slides (using Euparal). The studied material is deposited in the entomological collection of Estonian University of Life Sciences (**IZBE**) and the soil biology collection of Tallinn University of Technology (**TTUSB**), both in Tartu, Estonia. Various keys for Central, Northern, and East, European myriapods were used for identification (Schubart 1934, Lokshina 1969, Zalesskaya 1978, Blower 1985, Andersson et al. 2005, Bonato et al. 2005, Barber 2009).

## Results

All available material consisting of 1656 centipede, 4095 millipede, 29 symphylan, and six pauropod specimens were identified or re-identified and databased. The following list contains all the known published records of Estonian myriapods, followed by numbers of studied specimens and collecting localities. Full details for one finding from each locality are given in “Supplementary information”. Only publications with original data are listed, subsequent ones citing these (e.g., Schubart 1934, Lang 1954, Stojałowska 1961, Lokshina 1969, Atlavinytė and Lokshina 1971, Blower 1985, Spuņģis 2010) are omitted. An asterisk (*) marks previously unpublished species. The full list of records with all details will be available through the Estonian eBiodiversity portal (http://elurikkus.ut.ee; Abarenkov et al. 2010) and Global Biodiversity Information Facility (http.//www.gbif.org). The nomenclature and synonymies follow the ChiloBase (Bonato et al. 2016), “Atlas of European millipedes” (Kime and Enghoff 2011, 2017) and McAlpine and Shear (2018) for centipedes and millipedes, respectively, and the “Catalogue of Myriapoda in the Nordic Countries” (Andersson et al. 2008, 2013) for Symphyla and Pauropoda. For each species, a brief overview of its distribution is given (with emphasis on North-Eastern Europe).

**Table 1.** Collecting localities of Estonian myriapods. The localities’ numbers correspond to those on Figure 1. Localities within a range of less than 10 km are presented by one number, the different place names (sub-localities) under one number are designated consecutive letters (the coordinates apply only to the first of them).

No	Latitude / Longitude	Name
1	58.3300N, 21.9627E	a Kuusnõmme, b Eeriksaare, c Atla
2	58.4467N, 21.9391E	a Kõruse, b Undva, c Tagamõisa, d Neeme, e Tammese
3	57.9777N, 21.9971E	Türju
4	58.2467N, 22.0311E	Kipi
5	58.3909N, 22.0051E	a Oju, b Vilsandi,
6	57.9095N, 22.0552E	Sõrve peninsula
7	58.2833N, 22.1000E	a Audaku, b Sutru, c Kivesselja, d Pätsumaa bog, e Pitkasoo, f Suurissoo hill, g Surnuaiamägi, h Nakimetsa, i Suurmägi, j Laasma, k Viidumägi, l Upsi
8	58.3163N, 22.0806E	a Kanna, b Viidu
9	58.1234N, 22.1946E	Lõu
10	58.5105N, 22.2330E	Kugalepa
11	58.3188N, 22.3066E	a Mõnnuste, b Paadla
12	58.2209N, 22.2752E	Kaalupi
13	58.9414N, 22.4362E	Paope
14	58.3986N, 22.1278E	Viidumäe, Jürna liivad
15	58.2424N, 22.4246E	Suurlaht
16	58.1453N, 22.0570E	Abruka
17	58.3005N, 22.6459E	a Ilpla, b Kudjape
18	58.2256N, 22.6885E	Vanamõisa
19	58.3725N, 22.6697E	a near Kaali lake, b Võrsna
20	58.4563N, 22.7076E	Tika
21	58.5397N, 22.7307E	Õeste
22	58.7962N, 22.7555E	a Reigilaid, b Kassari
23	58.8289N, 22.9746E	a Suur-Pihlakare, b Öakse, c Saarnaki, d Aruküla, e Heltermaa, f Sarve
24	58.7956N, 23.0063E	a Saarnaki, b Hanikatsi, c Langekare
25	58.7421N, 23.1349E	Ahelaid
26	58.4828N, 22.9800E	a Koigi lake; b Koigi bog
27	58.5846N, 23.0246E	Orinõmme
28	58.4394N, 23.0680E	Asva
29	58.5506N, 23.1319E	Orissaare
30	58.6114N, 23.0911E	a Koguva, b Lepanina
31	58.6406N, 23.1588E	a Paenase, b Nõmmküla, c Üügu, d Lõetsa
32	58.4568N, 23.2673E	a Kahtla, b Kübassaare
33	58.5794N, 23.2709E	a Mäla, b Võiküla
34	58.9697N, 23.2058E	Vormsi: Kärret
35	59.0347N, 23.3047E	Vormsi: Diby
36	57.8062N, 23.2396E	Ruhnu
37	58.8377N, 23.3958E	Liialaid
38	58.6421N, 23.5167E	Hanila
39	58.5609N, 23.5522E	a Puhtu, b Laelatu, c Pivarootsi
40	58.6918N, 23.5821E	a Salevere Salumägi, b Saastna, c Metsküla
41	58.7440N, 23.6719E	a Keemu, b Kirikuküla Tika, c Kirikuküla Allika, d Kirikuküla Ennu, e Viita, f Penijõe
42	58.7531N, 23.8465E	a Kloostri, b Kelu, c Rõude, d Kasari, e Kirbla
43	58.8089N, 23.7011E	Rannamõisa
44	58.9492N, 23.5681E	a Haapsalu, b Linnamäe
45	59.2049N, 23.5988E	Noarootsi
46	58.5336N, 23.8299E	Paaderma
47	59.0356N, 23.6382E	Ingiküla
48	59.0371N, 23.6609E	Niibi
49	58.4319N, 24.0003E	Tõhela
50	59.2594N, 23.8737E	Vihterpalu
51	58.3144N, 23.9850E	a Tõstamaa, b Suti
52	58.5380N, 24.0062E	Nedrema
53	58.6455N, 24.1254E	Kurese
54	58.8075N, 24.0094E	Patsu fen
55	58.9020N, 24.0284E	a Marimetsa NR, b Kullamaa
56	58.9972N, 24.0562E	Risti
57	58.7757N, 24.2498E	Vana-Vigala
58	59.0723N, 24.2934E	Turba bog
59	59.3315N, 24.3745E	Tõmmiku
60	58.8958N, 24.3769E	a Sõtke, b Valgu, c Raela
61	58.7805N, 24.5625E	Inda
62	58.3884N, 24.5093E	Pärnu
63	58.1369N, 24.5141E	a Tolkuse bog, b Uulu,
64	58.0996N, 24.4737E	a Pulgoja, b Pikla, c Häädemeeste
65	58.0067N, 24.4423E	a Kabli b near Ikla
66	57.9947N, 24.5378E	Laulaste NR
67	58.2709N, 24.6411E	Laadi
68	59.3194N, 24.5581E	a Saue, b Pääsküla
69	59.3816N, 24.4628E	Vahi küla
70	59.3915N, 24.6434E	Vana-Mustamäe
71	59.2661N, 24.6483E	Kasemetsa-Kuresoo
72	59.5933N, 24.5025E	Naissaar
73	58.0783N, 24.8338E	Tali
74	58.0027N, 24.8769E	Sookuninga NR
75	58.0711N, 24.8608E	Kalita NR
76	58.9549N, 24.7641E	a Raela, b Varbola
77	59.0725N, 24.8077E	Hagudi
78	58.8879N, 24.6391E	Loe
79	58.6328N, 24.7013E	Lehu bog
80	58.9742N, 24.7021E	Kuusiku; Keo
81	58.8983N, 24.7616E	a Kõnnu, b Lellepere
82	59.5297N, 24.8577E	Lubja
83	59.2377N, 24.9311E	2km SE of Sõmeru
84	58.9459N, 25.1025E	Loosalu
85	58.7080N, 24.8780E	a Kõnnu bog, b Luuri bog
86	59.4630N, 24.9377E	Maardu
87	59.2781N, 25.6212E	Aegviidu
88	59.5084N, 25.5925E	Uuri
89	59.5841N, 25.6263E	Hara island
90	58.8165N, 25.1625E	Käru
91	58.6396N, 25.3039E	a Ramussaare, b Pikkmetsa, c Tõrvaaugu
92	58.2719N, 25.1798E	Riimaru
93	58.8099N, 25.3394E	Lokuta
94	59.0835N, 25.4052E	a Mustla, b Mustla Pühajärv
95	58.6333N, 25.5500E	a Võhma, b Koksvere
96	58.3593N, 25.5950E	Viljandi
97	58.0818N, 25.5253E	Viivre
98	59.6991N, 25.0211E	Keri island
99	58.7259N, 25.6007E	a Retla, b Kabala
100	58.6475N, 25.6717E	a Arussaare, b Kirivere, c Järavere
101	59.6049N, 25.9229E	Käsmu
102	59.5778N, 25.9556E	Võsu
103	59.4481N, 26.0126E	Viitna
104	59.4484N, 26.0118E	Koljaku-Oandu NR
105	59.5166N, 25.9746E	Palmse
106	59.5660N, 26.0880E	a Oandu, b Vihula
107	59.5557N, 26.3533E	Rutja; Varangu
108	58.9765N, 26.0454E	Koeru
109	59.0232N, 26.2443E	a Kamariku, b Rakke
110	58.8839N, 26.0433E	Sopaalliku
111	58.1413N, 25.6803E	Muti NR
112	58.0180N, 25.8794E	Helme
113	58.8457N, 26.2919E	a Kärde hill, b Kaera, c Pedja
114	58.0063N, 26.0553E	Soontaga NR
115	58.2388N, 26.1770E	a Rannu, b 2 km SW of Rannu
116	57.9127N, 26.1883E	Õru
117	58.1803N, 26.4205E	Elva-Vitipalu NR
118	58.2386N, 26.4433E	a Peedu, b Vapramäe
119	58.0399N, 26.2073E	Prange
120	58.0533N, 26.4898E	Otepää
121	58.3808N, 26.6222E	a Rahinge, b Tiksoja, c Tähtvere bog, d Õssu, e Tartu Eerika, f Merimetsa, g Tartu Tähtvere, h Kõrveküla
122	58.2301N, 26.7010E	Kambja
123	59.1857N, 26.1980E	a Porkuni, b Lasila
124	59.0373N, 26.6758E	between Venevere and Arukse
125	59.0115N, 26.4265E	Karaski
126	58.9092N, 26.5046E	a Pedjaääre, b Tudusoo NR
127	59.1527N, 26.8213E	Suigu NR
128	57.6878N, 26.1854E	Vaitka
129	57.6049N, 26.2749E	Koiva wooded meadow, b Koivakonnu, c Taheva
130	57.7522N, 26.4926E	a Karula Mähkli, b Küünimetsa
131	57.5727N, 26.6413E	Mõisamõtsa NR
132	57.6938N, 26.8850E	Saarlasõ küla
133	57.8386N, 27.0505E	Võrusoo
134	57.7355N, 27.0627E	Haanja NR
135	57.9422N, 27.4058E	a Rebasemäe, b Ilumetsa
136	57.8433N, 27.4626E	Piusa
137	58.1287N, 27.4990E	Räpina
138	57.8168N, 27.5180E	Obinitsa
139	58.0911N, 26.9050E	Palojärv
140	58.0524N, 27.0286E	Puuri
141	58.1514N, 26.8731E	a Voorepalu, b Ihamaru NR
142	58.1777N, 27.1467E	Mooste
143	58.2781N, 27.3210E	Järvselja
144	58.3287N, 26.9892E	Melliste
145	58.5170N, 26.9223E	Konnamõisa
146	58.5633N, 26.8772E	a Välgi NR, b Särgla, c Pataste
147	58.6032N, 27.1301E	Alatskivi
148	58.6558N, 26.9469E	a Pala, b Padakõrve NR
149	58.7296N, 26.8244E	a Odivere, b Maarja-Magdaleena
150	58.7394N, 26.9452E	Jõeääre
151	58.7430N, 26.8888E	a Ruskavere. b Võtikvere NR
152	58.7841N, 26.9330E	Nõmme
153	58.9636N, 26.8294E	a Kõveriku, b Avinurme
154	59.0230N, 27.0591E	Tudulinna
155	58.9656N, 27.0303E	Lohusuu
156	59.1732N, 26.9438E	Kaukvere
157	59.4443N, 26.9047E	Aseri taga
158	59.3588N, 26.9238E	Kiviõli
159	59.1644N, 27.0133E	Muraka NR
160	59.3179N, 27.1235E	Aidu
161	59.0894N, 27.1550E	Muraka NR
162	59.3858N, 27.2218E	Kohtla-Järve
163	59.2289N, 27.3247E	Mäetaguse NR
164	59.4439N, 27.3350E	a Valaste falls, b 5 km W of Toila
165	59.3948N, 27.3408E	Kukruse
166	59.4302N, 27.3900E	Toila
167	59.1523N, 27.3889E	Jõuga
168	59.0711N, 27.6277E	Agusalu LKA
169	59.0767N, 27.7033E	Permisküla
170	59.2384N, 27.8377E	Narva
171	59.1719N, 27.7961E	a Poruni, b Gorodenka

### 1. Chilopoda

#### 1.1. Geophilomorpha

##### 1.1.1. Geophilidae

*Geophiluscarpophagus Leach, 1814

Fig. 2(1)

**Studied material.** 2 specimens from 2 localities.

**General distribution.** Western Palaearctic species (Andersson et al. 2005), present also in southern Sweden and south-western Finland (Andersson et al. 2008), Latvia (Bonato et al. 2005) and Lithuania (Tuf et al. 2015).

**Comments.** The species is rare in Estonia.

***Geophiluselectricus* (Linnaeus, 1758)**

Fig. 2(2)

**Literature sources.**Volkova 2016: 504.

**Studied material.** 5 specimens from 4 localities.

**Distribution.** Western Palaearctic species introduced also to North America (Andersson et al. 2005), present also in southern Sweden and south-western Finland (Andersson et al. 2008), Latvia (Bonato et al. 2005) and Lithuania (Tuf et al. 2015).

**Comments.** The species is rare in Estonia.

*Geophilusflavus (De Geer, 1778)

Figs 2(3), 6

**Literature sources.**Ivask et al. in press.

**Studied material.** 68 specimens from 24 localities.

**General distribution.** Western Palaearctic species introduced also to North America (Andersson et al. 2005), widespread in Scandinavia and Finland (Andersson et al. 2008), Latvia (Bonato et al. 2005) and Lithuania (Tuf et al. 2015).

**Comments.** A common species in different habitats, but absent in wet areas.

***Geophilusproximus* C.L. Koch, 1847**

Figs 2(4), 6

**Literature sources.**Riikoja 1955: 15, Ivask 2011: 2, Ivask et al. in press.

**Studied material.** 57 specimens from 26 localities.

**General distribution.** Central and North European species, (Andersson et al. 2005), widespread in Scandinavia and Finland (Andersson et al. 2008), Latvia (Bonato et al. 2005) and Lithuania (Tuf et al. 2015).

**Comments.** A common species in different habitats, but absent in wet areas.

***Geophilustruncorum* Bergsoe & Meinert, 1866**

Figs 2(5), 6

**Literature sources.**Vilbaste 1970: 174, Vilbaste et al. 1985: 152, Vilbaste and Vilbaste 1993: 319 [as: *Brachygeophilustruncorum* Mnr.], Ivask et al. in press.

**Studied material.** 66 specimens from 27 localities.

**General distribution.** Western Palaearctic species, present also in southern Sweden and south-western Finland (Andersson et al. 2008), Latvia (Bonato et al. 2005) and Lithuania (Tuf et al. 2015).

**Comments.** A common species in different habitats, especially in soil samples.

*Stenotaenialinearis (C.L.Koch, 1835)

Fig. 2(7)

**Studied material.** 5 specimens from 1 locality.

**General distribution.** Western Palaearctic species, exclusively synanthropic in northern Europe, present also in Latvia (Bonato et al. 2005) and Finland (Andersson et al. 2008).

**Comments.** The species was recently shown to comprise several cryptic lineages (Wesener et al. 2015). It is probably an introduced species in Estonia (only found in the Tartu Botanical Garden in Estonia and present also only synanthropically in the neighbouring countries).

***Pachymeriumferrugineum* (C. L. Koch, 1835)**

Figs 2(6), 6

**Literature sources.**Riikoja 1955: 15, Vilbaste 1970: 174, Vilbaste et al. 1985: 152, Ivask et al. in press.

**Studied material.** 31 specimens from 14 localities.

**General distribution.** Holarctic species (Andersson et al. 2005), widespread in Sweden and Finland (Andersson et al. 2008), Latvia (Bonato et al. 2005) and Lithuania (Tuf et al. 2015).

**Comments.** The species is more common in coastal areas and rare elsewhere. It seems to favour dry habitats.

##### 1.1.2 Linotaeniidae

*Strigamiatranssilvanica (Verhoeff, 1928)

Fig. 2(8)

**Studied material.** 3 specimens from 2 localities.

**General distribution.** Mainly a Central European species but recently found also in Latvia (Bonato et al. 2005). The species has no published records from north-western Russia (Volkova 2016), but there is a specimen collected from Izborsk (Pskov region, 10 km of Estonian border) in the IZBE collection.

**Comments.** The species is rare in Estonia. Both findings are from human settlements.

##### 1.1.3. Schendylidae

***Schendylanemorensis* (C.L. Koch, 1837)**

Fig. 3(5)

**Literature sources.**Riikoja 1955: 15, Ivask et al. in press.

**Studied material.** 19 specimens from 12 localities.

**General distribution.** Western Palaearctic species (Barber 2009), introduced to North America, present also in southern Sweden and south-western Finland (Andersson et al. 2008), Latvia (Bonato et al. 2005), and Lithuania (Tuf et al. 2015).

**Comments.** More common in western Estonia, found mainly in soil samples.

#### 1.2. Lithobiomorpha

##### 1.2.1. Henicopidae

***Lamyctesemarginatus* (Newport 1844)**

Fig. 2(9)

**Literature sources.**Riikoja 1955: 15 [as: *L.fulvicornis* Meinert], Ivask 2011: 1.

**Studied material.** 7 specimens from 4 localities.

**General distribution.** A semi-cosmopolitan species widespread also in Scandinavia and Finland (Andersson et al. 2008).

**Comments.** Locally common in western Estonia, not found elsewhere.

##### 1.2.2. Lithobiidae

**Lithobius (Lithobius) borealis Meinert, 1868**

Fig. 2(10)

**Literature sources.**Ivask 2011: 2.

**Studied material.** 11 specimens from 4 localities.

**General distribution.** Central and west-European species, present also in Sweden (Andersson et al. 2008), and Lithuania (Tuf et al. 2015).

**Comments.** More common in western Estonia, but nowhere abundant.

**Lithobius (Lithobius) erythrocephalus C. L. Koch, 1847**

Figs 2(13), 6

**Literature sources.**Riikoja 1955: 15, Vilbaste 1979: 99, Vilbaste et al. 1985: 152, Ivask 2011: 2, Ivask et al. in press.

**Studied material.** 141 specimens from 48 localities.

**General distribution.** Western Palaearctic species, widespread in Scandinavia and Finland (Andersson et al. 2008), Latvia (Trautberg 1929) and Lithuania (Tuf et al. 2015).

**Comments.** A common species in different habitats.

**Lithobius (Lithobius) forficatus (Linnaeus, 1758)**

Figs 2(14), 6

**Literature sources.**Riikoja 1955: 15, Vilbaste 1970: 173, Vilbaste et al. 1985: 152, Remm 1988: 128, Vilbaste and Vilbaste 1993: 319, Ivask 2011: 2, Kalda et al. 2015: 90, Ivask et al. in press.

**Studied material.** 352 specimens from 89 localities.

**General distribution.** Holarctic species, widespread in Scandinavia and Finland (Andersson et al. 2008), Latvia (Trautberg 1929) and Lithuania (Tuf et al. 2015), present also in Lenigrad region (north-western Russia) (Zalesskaya 1978).

**Comments.** One of the two most common centipede species in different habitats, but favours more xeric areas than *Lithobiuscurtipes*.

**Lithobius (Lithobius) lucifugus L. Koch 1862**

Fig. 2(15)

**Literature sources.**Vilbaste 1979: 99, Vilbaste et al. 1985: 152, Vilbaste and Vilbaste 1993: 319.

**Studied material.** 5 specimens from 4 localities.

**General distribution.** Central- and south-east European species, present in Latvia (Trautberg 1929), Lithuania (Tuf et al. 2015) and on the Swedish islands Öland and Gotland (Andersson et al. 2008).

**Comments.** The species is widespread but rare in Estonia.

*Lithobius (Lithobius) melanops Newport, 1845

Fig. 3(1)

**Studied material.** 7 specimens from 7 localities.

**General distribution.** Western Palaearctic species introduced to North America, present also in and Sweden (Andersson et al. 2008), Latvia (Trautberg 1929) and Lithuania (Tuf et al. 2015), synanthropic in southern Finland (Palmen 1949).

**Comments.** The species is widespread but infrequent in Estonia.

**Lithobius (Lithobius) pelidnus Haase, 1880**

Fig. 3(3)

**Literature sources.**Riikoja 1955: 15, Remm 1988: 128.

**Studied material.** 27 specimens from 4 localities.

**General distribution.** Central- and East-European species present also in southern Sweden (Andersson et al. 2008) and Lithuania (Tuf et al. 2015).

**Comments.** The species is widespread but infrequent in Estonia, found only in bogs and boreo-nemoral forests.

**Lithobius (Lithobius) tenebrosus Meinert, 1872**

Figs 3(4), 6

**Literature sources.** Riikoja, 1955: 15 [as *Lithobiusnigrifrons*], Ivask 2011: 1.

**Studied material.** 43 specimens from 25 localities.

**General distribution.** Western Palaearctic species common in Finland and Sweden (Andersson et al. 2005), Latvia (Trautberg 1929) and Lithuania (Tuf et al. 2015).

**Comments.** A common species in different habitats, but avoids human settlements. H. Lohmander (1948) described a subspecies fennoscandicusLohmander 1948 from Scandinavia, the description of which Estonian specimens generally match, but as the main subspecific difference concern colouration, more fresh specimens need to be studied.

**Lithobius (Monotarsobius) crassipes C.L. Koch, 1862**

Figs 2(12), 6

**Literature sources.**Riikoja 1955: 15, Vilbaste 1979: 99, Vilbaste et al. 1985: 152, Remm 1988: 128, Vilbaste and Vilbaste 1993: 319, Ivask et al. in press.

**Studied material.** 25 specimens from 18 localities.

**General distribution.** Palaearctic species, present also in southern Finland, Sweden (Andersson et al. 2008) and Lithuania (Tuf et al. 2015).

**Comments.** The species is widespread but infrequent in Estonia, avoids wet habitats.

**Lithobius (Monotarsobius) curtipes C.L. Koch, 1847**

Figs 2(11), 6

**Literature sources.**Riikoja 1955: 15; Vilbaste 1970: 173; Vilbaste 1979: 99; Vilbaste et al. 1985: 152, Remm 1988: 128, Vilbaste and Vilbaste 1993: 319, Ivask 2011: 1, Kalda et al. 2015: 90, Ivask et al. in press.

**Studied material.** 730 specimens from 106 localities.

**General distribution.** Mainly a central and East European species, common in Sweden and Finland (Andersson et al. 2005, Palmén 1948), Latvia (Trautberg 1929) and Lithuania (Tuf et al. 2015).

**Comments.** One of the two most common centipede species in different habitats, favours more fresh habitats than *L.forficatus*.

*Lithobius (Sigibius) microps Meinert, 1868

Fig. 3(2)

**Literature sources.**Ivask et al. in press.

**Studied material.** 52 specimens from 17 localities.

**General distribution.** A western Palaearctic species, introduced to North America, also present in Finland, Sweden (Andersson et al. 2005), and Lithuania (Tuf et al. 2015).

**Comments.** The species is widespread but infrequent in Estonia, seems to avoid wet habitats.

### 2. Diplopoda

#### 2.1. Polyxenida

##### 2.1.1. Polyxenidae

***Polyxenuslagurus* Linnaeus, 1758**

Fig. 3(6)

**Literature sources.**Palmén 1949: 4, Vilbaste 1953: 16, Vilbaste 1970: 174, Remm 1988: 128, Kime and Enghoff 2011: 21.

**Studied material.** 27 specimens from 8 localities.

**General distribution.** Holarctic species, present in Latvia (Spuņģis 2010), Lithuania (Atlavinytė and Lokshina 1971), Sweden, Finland (Andersson et al. 2008) and Leningrad region (north-western Russia) (Lokshina 1969).

**Comments.** The species is common in soil near seashore in Western Estonia, but rare and saproxylic inland. It may be more widespread, but underdetected due to its small size.

#### 2.2. Polyzoniida

##### 2.2.1. Polyzoniidae

***Polyzoniumgermanicum* Brandt, 1837**

Fig. 3(7)

**Literature sources.**Schubart 1930: 193, Vilbaste 1953: 45, Vilbaste 1970: 174, Vilbaste 1979: 99, Vilbaste et al. 1985: 152, Remm 1988: 128, Vilbaste and Vilbaste 1993: 319, Kime and Enghoff 2011: 42.

**Studied material.** 26 specimens from 16 localities.

**General distribution.** Western Palaearctic species, present in Latvia (Spuņģis 2010), Sweden, and Finland (Andersson et al. 2008).

**Comments.** The species is widespread but infrequent in different habitats, favours more fresh habitats and has not been found in human-disturbed areas.

#### 2.3. Chordeumatida

##### 2.3.1. Craspedosomatidae

*Craspedosomaraulinsii Leach, 1814

Figs 3(8), 7

**Studied material.** 277 specimens from 23 localities.

**General distribution.** Western Palaearctic species introduced also to North America, present in Latvia (Spuņģis 2010), Lithuania (Atlavinytė and Lokshina 1971), Sweden, and Finland (Andersson et al. 2008).

**Comments.** This represents the first formal record of the species in Estonia after its mention by Saar and Takkis (2010) in the popular journal GEO. The species is widespread but favours fresh habitats. It seems to have recently colonised Estonia (but see Discussion).

##### 2.3.2 Mastigophorophyllidae

***Mastigophorophyllonsaxonicum* Verhoeff, 1916**

Fig. 3(9)

**Literature sources.**Schubart 1930: 193, Vilbaste 1953: 18.

**General distribution.** Central European species absent from Scandinavia and Finland (Andersson et al. 2008, 2013), present in Lithuania (Atlavinytė and Lokshina 1971) formerly found from Latvia, but not recently recorded (Spuņģis 2010).

**Comments.** The species was described as frequent in southern Estonia (Schubart 1930) but there are no recent records since Vilbaste 1953. The species may have become more rare or extinct in Estonia. Schubart (1930) mentioned a few exact localities in his work, viz. Sõrve peninsula, Abruka Island, the vicinity of Pärnu and Vilbaste (1953) repeats these data and adds Tartu as the northern boundary of its range. All these localities were studied in the current research but the species was not found. It is possible that the record from Tartu refers instead to Haase’s specimen of Craspedosomamutabilevar.fasciatum, interpreted as a misidentification of *M.saxonicum* by Schubart (1930). Vilbaste does not mention the species (nor any other Chordeumatids) in any of his later works.

#### 2.4. Polydesmida

##### 2.4.1. Polydesmidae

***Brachydesmussuperus* Latzel, 188**4

Fig. 3(11)

**Literature sources.**Schubart 1930: 193, Vilbaste 1953: 19, Vilbaste 1970: 174, Ivask et al. in press.

**Studied material.** 14 specimens from 8 localities.

**General distribution.** Western Palaearctic species, introduced also to many other parts of the world, present in Latvia (Spuņģis 2010), Lithuania (Atlavinytė and Lokshina 1971), Sweden, and Finland (Andersson et al. 2008).

**Comments.** The species is widespread, but infrequent, in different habitats.

***Polydesmuscomplanatus* (Linnaeus, 1761)**

Figs 3(12), 7

**Literature sources.**Schubart 1930: 193, Vilbaste 1953: 20, Vilbaste et al. 1985: 152, Remm 1988: 128, Kime and Enghoff 2011: 62.

**Studied material.** 59 specimens from 24 localities.

**General distribution.** Western Palaearctic species, introduced also to North America, present in Latvia (Spuņģis 2010), Lithuania (Atlavinytė and Lokshina 1971), Sweden, Finland (Andersson et al. 2008), and Leningrad region (north-western Russia) (Lokshina 1969).

**Comments.** The species is widespread and common, in different habitats.

***Polydesmusdenticulatus* C.L. Koch, 184**7

Figs 3(13), 7

**Literature sources.**Schubart 1930: 193, Vilbaste 1953: 21, Vilbaste 1970: 174, Vilbaste 1979: 99, Vilbaste et al. 1985: 152, Kime and Enghoff 2011: 63, Kalda et al. 2015: 90, Ivask et al. in press.

**Studied material.** 796 specimens from 82 localities.

**General distribution.** Western Palaearctic species, introduced also to North America, common in Latvia (Spuņģis 2010), Lithuania (Atlavinytė and Lokshina 1971), Sweden, Finland (Andersson et al. 2008), Leningrad, and Pskov regions (north-western Russia) (Lokshina 1969).

**Comments.** The species is widespread and common, in different habitats.

***Polydesmusinconstans* Latzel, 1884**

Figs 3(14), 7

**Literature sources.**Schubart 1930: 193 [as *Polydesmuscoriaceus*], Vilbaste 1953: 22, Vilbaste 1970: 174, Remm 1988: 128, Kime and Enghoff 2011: 65, Ivask et al. in press.

**Studied material.** 29 specimens from 13 localities.

**General distribution.** Western Palaearctic species, introduced also to North America, present in Latvia (Spuņģis 2010), Lithuania (Atlavinytė and Lokshina 1971), Sweden, and Finland (Andersson et al. 2008).

**Comments.** The species is widespread but infrequent, in different habitats, but prefers woodlands. There has been a confusion of this species with Western European *P.coriaceus* Porat, 1871 (cf. Vilbaste 1953, Blower 1985, Spuņģis 2010). The record of *P.coriaceus* in a posthumously published work by J. Vilbaste (Vilbaste and Vilbaste 1993) is inexplicable and obviously erroneous. The Estonian specimens identified as *P.coriaceus* in Zoologische Staatssammlung München, Germany (Verhoeff collection, collected from Tallinn, no date) also belong to *P.inconstans* (J. Spelda, pers. comm.).

#### 2.5. Julida

##### 2.5.1. Blaniulidae

***Blaniulusguttulatus* (Fabricius, 1798)**

Fig. 3(15)

**Literature sources.**Schubart 1930: 193, Kaarep et al. 1949: 176, Vilbaste 1953: 29, Kime and Enghoff 2017: 27, Ivask et al. in press.

**Studied material.** 35 specimens from 6 localities.

**General distribution.** Western Palaearctic species, introduced also to many other parts of the world, present in Latvia (Spuņģis 2010), Lithuania (Atlavinytė and Lokshina 1971), Sweden, Finland (Andersson et al. 2008) and Leningrad region (north-western Russia) (Lokshina 1969).

**Comments.** The species is widespread but infrequent, mostly synanthropic.

***Boreoiulustenuis* (Bigler, 1913)**

Fig. 4(1)

**Literature sources.**Vilbaste 1953: 29, Ivask et al. in press.

**Studied material.** 7 specimens from 4 localities.

**General distribution.** Northern and central European species, present in Latvia (Spuņģis 2010), Finland (Andersson et al. 2008), and Leningrad region (north-western Russia) (Lokshina 1969).

**Comments.** The species is widespread but rare.

***Nopoiuluskochii* (Gervais, 1847)**

Fig. 4(2)

**Literature sources.**Schubart 1930: 193 [as *Nopoiulusarmatus*], Vilbaste 1953: 26, Vilbaste 1970: 174 [as *Nopoiulusvenustus*], Kime and Enghoff 2017: 33.

**Studied material.** 7 specimens from 3 localities.

**General distribution.** Western Palaearctic species, introduced also to many other parts of the world, present in Latvia (Spuņģis 2010), Lithuania (Atlavinytė and Lokshina 1971), Sweden, Finland (Andersson et al. 2008), and Leningrad region (north-wWestern Russia) (Lokshina 1969).

**Comments.** The species is rare, found only in northern and western Estonia.

***Proteroiulusfuscus* (Am Stein, 1857)**

Figs 4(3), 7

**Literature sources.**Schubart 1924: 57 [as *Nopoiuluspalmatuscaelebs*], Schubart 1930: 193, Vilbaste 1953: 27, Vilbaste 1970: 174, Vilbaste et al. 1985: 152, Remm 1988: 128, Vilbaste and Vilbaste 1993: 319, Kime and Enghoff 2017: 34, Ivask et al. in press.

**Studied material.** 239 specimens from 42 localities.

**General distribution.** Western Palaearctic species, introduced also to North America, present in Latvia (Spuņģis 2010), Lithuania (Atlavinytė and Lokshina 1971), Sweden, Finland (Andersson et al. 2008), Leningrad, and Novgorod regions (north-western Russia) (Lokshina 1969).

**Comments.** The species is widespread and common, especially in moist habitats, usually associated with decaying wood.

##### 2.5.2. Nemasomatidae

***Nemasomavaricorne* C. L. Koch, 1847**

Figs 3(10), 7

**Literature sources.**Vilbaste 1953: 25 [as *Isobatesvaricornis*], Kime and Enghoff 2017: 199, Ivask et al. in press.

**Studied material.** 126 specimens from 28 localities.

**General distribution.** Central and East-European species, present in Latvia (Spuņģis 2010), Lithuania (Atlavinytė and Lokshina 1971), Sweden and Finland (Andersson et al. 2008).

**Comments.** The species is widespread and common in different types of woodland. Climbs also in trees (as several individuals were found in trunk window traps).

##### 2.5.3. Julidae

***Allajulusnitidus* (Verhoeff, 1891)**

Fig. 4(4)

**Literature sources.**Ivask 2011: 1, Ivask et al. in press.

**Studied material.** 38 specimens from 14 localities.

**General distribution.** Central and northern European species, not found in Latvia (Spuņģis 2010) and Finland, present in Sweden (Andersson et al. 2008, 2013).

**Comments.** The species is widespread but frequent only in western Estonia, mostly associated with open landscape.

***Brachyiuluspusillus* (Leach, 1814)**

Fig. 4(5)

**Literature sources.**Schubart 1930: 193 [as *Brachyiuluslittoralis*], Vilbaste 1953: 42 [as *Brachyiuluslittoralis*], Kime and Enghoff 2017: 47.

**General distribution.** Western Palaearctic species introduced to many parts of the world, rare in Latvia (Spuņģis 2010), present in Lithuania (Atlavinytė and Lokshina 1971) and southern Sweden, not found in Finland (Andersson et al. 2008, 2013).

**Comments.** No specimens were collected during our studies or are preserved in Estonian collections. The current status of the species in Estonia is unclear as it has been reported as rare also in the past. It seems that both Schubart (1930) and Vilbaste (1953) refer to the same single specimen (loc. 20). There appears to be another finding from Hiiumaa Island according to Kime and Enghoff (2017), but we failed to trace the origin of that record (H. Enghoff, pers. comm.). It is not impossible that the record from Hiiumaa is a misinterpretation of the historical place name Tickhof, which is present also on Hiiumaa (Kongo 2016), but Schubart states the locality as “*in einem Garten in Tickhof auf Ösel*” - “in a garden in Tickhof on Ösel (=Saaremaa island)”, and Vilbaste (1953) repeats that almost literally.

*Cylindroiulusbritannicus (Verhoeff, 1891)

Fig. 4(6)

**Studied material.** 10 specimens from 2 localities.

**General distribution.** Western Palaearctic species, introduced to many parts of the world, present in Latvia (Spuņģis 2010), Lithuania (Atlavinytė and Lokshina 1971), Sweden and Finland (Andersson et al. 2008).

**Comments.** The species is rare, found only on western Estonian islands.

***Cylindroiuluscaeruleocinctus* (Wood, 1864)**

Figs 4(8), 7

**Literature sources.**Schubart 1930: 193 [as *Cylindroiulusteutonicus*], Kaarep et al. 1949: 176 [as *Cylindroiulusteutonicus*], Vilbaste 1953: 33 [as *Cylindroiulusteutonicus*], Kime and Enghoff 2017: 55.

**Studied material.** 237 specimens from 31 localities.

**General distribution.** Western Palaearctic species, introduced to North America, present in Latvia (Spuņģis 2010), Lithuania (Atlavinytė and Lokshina 1971), Sweden, Finland (Andersson et al. 2008), Leningrad, and Pskov regions (north-western Russia) (Lokshina 1969).

**Comments.** The species is widespread and common, especially in or close to human settlements. The records of *Ophyiuluspilosus* (Newport, 1842) as a pest of potatoes in Estonia (Zolk 1923) probably refer to this species instead (Vilbaste 1953).

***Cylindroiuluslatestriatus* (Curtis, 1845)**

Fig. 4(7)

**Literature sources.**Schubart 1930: 193 [as *Cylindroiulusfrisius*], Vilbaste 1953: 35 [as *Cylindroiulusfrisius*], Kime and Enghoff 2017: 63.

**Studied material.** 44 specimens from 14 localities.

**General distribution.** Western Palaearctic species, introduced to many parts of the world, present in Latvia (Spuņģis 2010), Lithuania (Atlavinytė and Lokshina 1971), Sweden, Finland (Andersson et al. 2008), and Leningrad region (north-western Russia) (Lokshina 1969).

**Comments.** The species is widespread but infrequent, more common in western Estonia. It seems to prefer drier habitats.

***Julusscandinavius* Latzel, 1884**

Fig. 4(9)

**Literature sources.**Ivask 2011: 2, Ivask et al. in press.

**Studied material.** 17 specimens from 7 localities.

**General distribution.** Central and northern European species, not found in Latvia (Spuņģis 2010) and in Finland, present in southern Sweden (Andersson et al. 2008).

**Comments.** The species is widespread but infrequent, most findings are from Western Estonia.

***Julusscanicus* Lohmander, 1925**

Figs 4(10), 7

**Literature sources.**Kaarep et al. 1949: 176, Vilbaste 1953: 38, Ivask 2011: 2, Ivask et al. in press.

**Studied material.** 252 specimens from 22 localities.

**General distribution.** Mainly a Central European species, present in Latvia (Spuņģis 2010), and southern Sweden, not found in Finland (Andersson et al. 2008, 2013).

**Comments.** The species is common in western Estonia, but not found elsewhere.

***Julusterrestris* Linnaeus, 1758**

Figs 4(11), 7

**Literature sources.**Schubart 1930: 193, Vilbaste 1953: 39, Vilbaste 1970: 174, Vilbaste et al. 1985: 152, Kime and Enghoff 2017: 96, Ivask et al. in press.

**Studied material.** 304 specimens from 34 localities.

**General distribution.** Mainly a central European species, present in Latvia (Spuņģis 2010), Lithuania (Atlavinytė and Lokshina 1971), Sweden, and Finland (Andersson et al. 2008).

**Comments.** The species is frequent in western Estonia, but rare elsewhere. Clearly prefers open landscapes.

***Leptoiuluscibdellus* (Chamberlin 1921)**

Figs 4(12), 7

**Literature sources.**Schubart 1930: 193 [as *Leptoiulusminutus*], Vilbaste 1953: 40 [as *Leptoiulusminutus*], Ivask 2011: 1, Kime and Enghoff 2017: 101, Ivask et al. in press.

**Studied material.** 274 specimens from 30 localities.

**General distribution.** Central European species, present in Latvia (Spuņģis 2010), Lithuania (Atlavinytė and Lokshina 1971), Sweden, and Finland (Andersson et al. 2008).

**Comments.** The species is widespread, but common only in western Estonia. Clearly prefers open landscapes.

***Leptoiulusproximus* (Němec, 1896)**

Figs 4(13), 8

**Literature sources.**Schubart 1924: 57 [as *Leptoiulusbuckkensis*], Schubart 1930: 193 [as *Leptoiulusbuekkensis*], Vilbaste 1953: 39, Vilbaste 1970: 174, Vilbaste 1979: 99, Vilbaste et al. 1985: 152, Remm 1988: 128, Kime and Enghoff 2017: 250, Ivask et al. in press.

**Studied material.** 584 specimens from 81 localities.

**General distribution.** present in Latvia (Spuņģis 2010), Lithuania (Atlavinytė and Lokshina 1971), Sweden, Finland (Andersson et al. 2008), and Leningrad region (north-western Russia) (Lokshina 1969).

**Comments.** The species is widespread and very common in different habitats, with a slight preference to open landscape. The report of *Ophyiuluspilosus* in Estonia (Ivask 2011) has proved erroneous after re-examining the material, and belongs also to this species.

***Megaphyllumsjaelandicum* (Meinert, 1868)**

Figs 4(14), 8

**Literature sources.**Schubart 1930: 193 [as *Chromatoiulussjaelandicus*], Vilbaste 1953: 43 [as *Chromatoiulussjaelandicus*], Vilbaste et al. 1985: 152, Remm 1988: 128, Lazányi and Vagalinski 2013: 86, Kime and Enghoff 2017: 128.

**Studied material.** 80 specimens from 25 localities.

**General distribution.** Central and eastern Palaearctic species, present in Latvia (Spuņģis 2010), southern Sweden, Finland (Andersson et al. 2008), Leningrad and Novgorod regions (north-western Russia) (Lokshina 1969).

**Comments.** The species is widespread and common in different habitats, with a slight preference to fresh forests.

***Ommatoiulussabulosus* (Linnaeus, 1758)**

Figs 4(15), 8

**Literature sources.**Schubart 1930: 193 [as *Archiulussabulosus*], Vilbaste 1953: 45 [as *Schizophyllumsabulosum*], Vilbaste 1979: 99 [as *Schizophyllumsabulosum*], Remm 1988: 128 [as *Schizophyllumsabulosum*], Kalda et al. 2015: 90, Kime and Enghoff 2017: 276, Ivask et al. in press.

**Studied material.** 659 specimens from 84 localities.

**General distribution.** Western Palaearctic species, common in Latvia (Spuņģis 2010), Sweden, Finland (Andersson et al. 2008)m and Leningrad region (north-western Russia) (Lokshina 1969).

**Comments.** A very common species in different habitats. Climbs also in trees (as several individuals were found in trunk window traps). A mass outbreak of the species was observed near Ikla (south-western Estonia) in June 2018, where numerous specimens entered houses (see also Discussion).

***Rossiulusvilnensis* (Jawlowski, 1925)**

Fig. 5(1)

**Literature sources.**Schubart 1930: 193 [as *Archiulusvilnense*], Vilbaste 1953: 44 [as Schizophyllum (Sarmatiulus) vilnense], Vilbaste and Vilbaste 1993: 319, Kime and Enghoff 2017: 155.

**Studied material.** 16 specimens from 8 localities.

**General distribution.** Central and east-European species (Lokshina 1969), present in Lithuania (Atlavinytė and Lokshina 1971) rare in Latvia (Spuņģis 2010), not found in Finland and Sweden (Andersson et al. 2008, 2013).

**Comments.** The species is widespread but infrequent, not found from northern Estonia.

***Uncigerfoetidus* C.L. Koch, 1838**

Figs 5(2), 8

**Literature sources.**Schubart 1930: 193, Vilbaste 1953: 41, Vilbaste 1979: 99, Kime and Enghoff 2017: 168.

**Studied material.** 728 specimens from 18 localities.

**General distribution.** Western Palaearctic species, present in Latvia (Spuņģis 2010), Lithuania (Atlavinytė and Lokshina 1971), Sweden, southern Finland (Andersson et al. 2008).

**Comments.** The species is widespread and common, in different habitats, but avoids very wet ones and seems to be favoured by human influence.

***Xestoiuluslaeticollis* (Porat, 1889)**

Fig. 5(3)

**Literature sources.**Schubart 1930: 193 [as *Microiuluslaeticollismierzejewskii*], Vilbaste et al. 1985: 38 [as *Microiuluslaeticollismierzejewskii*], Remm 1988: 128, Vilbaste and Vilbaste 1993: 319, Kime and Enghoff 2017: 171, Ivask et al. in press.

**Studied material.** 19 specimens from 13 localities.

**General distribution.** Central and east European species, present in Lithuania (Atlavinytė and Lokshina 1971), Latvia (Spuņģis 2010) and southern Sweden, not found in Finland (Andersson et al. 2008, 2013).

**Comments.** The species is infrequent and found only from Western Estonia.

### 3. Symphyla

#### 3.1 Scutigerellidae

*Scutigerellaimmaculata (Newport, 1845)

Fig. 5(4)

**Studied material.** 14 specimens from 7 localities.

**General distribution.** Unclear, present in Finland and Sweden (Andersson et al. 2005, 2008, 2013) probably also in Latvia (Eglītis 1954).

#### 3.2 Scolopendrellidae

*Symphylellavulgaris (Hansen, 1903)

Fig. 5(5)

**Studied material.** 10 specimens from 6 localities.

**General distribution.** A widespread Holarctic species, present in Finland and Sweden (Andersson et al. 2008), possibly also in Latvia (*Symphylella* sp. in Eglītis 1954).

*Scolopendrellopsissubnuda (Hansen, 1903)

Fig. 5(6)

**Studied material.** 5 specimens from 2 localities.

**General distribution.** Western Palaearctic species, present also in Sweden and Finland (Andersson et al. 2005).

### 4. Pauropoda

We regard the record of *Pauropushuxleyi* Lubbock, 1867 near Narva (Schmidt 1894, Andersson et al. 2005: 274) being dubious, as it is unclear from which side of the current Estonian-Russian border it was collected and since several related species were undescribed at the time.

#### 4.1. Pauropodidae

*Decapauropuscuenoti (Remy, 1931)

Fig. 5(7)

**Studied material.** 5 specimens from 1 locality.

**General distribution.** Possibly a Holarctic species with predominantly a northern distribution, present also in Sweden and Finland (Andersson et al. 2005).

*Decapauropusgracilis (Hansen, 1902)

Fig. 5(8)

**Studied material.** 1 specimen from 1 locality.

**General distribution.** Possibly a Holarctic species with introductions to South Asia and South America, present also in Sweden and Finland (Andersson et al. 2005).

## Discussion

The current study adds six centipede and two millipede new country records, while two millipedes, viz. *Polydesmuscoriaceus* and *Ophyiuluspilosus* are presently removed from the Estonian checklist. All the Symphylan and Pauropod species represent new records. It is unclear whether the new records are due to insufficient previous data (which may well be true for centipedes, except *Stenotaenialinearis*) or range shifts. The human or climate driven range shifts up to over hundred km northwards in recent decades have been also detected elsewhere (David 2009). The changes in occurrence frequencies of the species that have been observed also in the neighbouring countries, e.g., in Finland by Lehtinen and Terhivuo (1996), concern *Cylindroiuluscaeruleocinctus* and *Uncigerfoetidus*. Both species were earlier reported as rare in Estonia (cf. Vilbaste 1953) but proved to be common and widespread after the present study. Mass outbreaks of some julid species have also spread northwards (Kania and Tracz 2005) and were recently seen in Lithuania e.g., *O.sabulosus* in 2015–2016 (J. Rimšaitė pers. comm.). This type of event is reported here for the first time in Estonia, with a localised outbreak of *O.sabulosus* in south-western Estonia, occurred in June 2018. The Estonian findings of *Lithobiuspelidnus*, *Strigamiatranssilvanica*, *Brachyiuluspusillus*, *Allajulusnitidus*, *Xestoiuluslaeticollis*, and *Rossiulusvilnense* represent the northernmost records for those species.

The range of *Craspedosomaraulinsii* seems to expand north and eastwards. It was apparently first collected in Latvia between 2003 and 2008 (Andersson et al. 2005, Spuņģis 2010), 2010 in Estonia and 2006 in Moscow region of Russia (Golovatch and Matyukhin 2011). The first record(s) from Finland seem to be probably from 2001 to 2005 (reported as present in southern Finland by Andersson et al. 2005, but absent according to Kime 2001). However, we failed to find the original source of the Finnish records (V. Huhta, H. Enghoff, P. Djursvoll, P. Cardoso pers. comm.). The actual appearance of *C.raulinsii* in the Northern Baltic region can be decades earlier, as the myriapod fauna of Estonia and Latvia was not systematically monitored in the 1980-s and 1990-s. On the other hand, if the record of Craspedosomamutabilevar.fasciatum (if a synonym of *C.raulinsii*, as in Sierwald and Spelda 2018) from Tartu (Haase 1886) is correct, the species distribution range may have fluctuated also in the past. The name is a synonym of *Mastigonabosniense* according to Schubart (1934), and the identity with that species is also possible, as a specimen of *Mastigona* sp. (as *Heteroporatia* sp.) has been found in Latvia (Becker 1929, Spuņģis 2010).

Four species, viz. *Strongylosomastigmatosum*, *Cylindroiuluspunctatus*, *Archiboreiuluspallidus* and *Choneiuluspalmatus*, occurring in neighbouring Latvia and/or Finland might be found also in Estonia, but more studies, especially in southern Estonia are needed. The species *Mastigophorophyllonsaxonicum*, previously reported from many localities in southern Estonia was not re-found, and appears to have become more rare or extinct (which is also the case in Latvia, Spuņģis 2010). Spuņģis (2010) discusses the possibility that the northern Baltic records of *M.saxonicum* are misidentifications of *C.raulinsii*, which seems improbable to us as both species should have been well known for O. Schubart. Several species e.g., *Lamyctesemarginatus*, *Pachymeriumferrugineum*, *Julusterrestris*, *Julusscandinavius*, *Allajulusnitidus*, *Leptoiuluscibdellus*, and *Xestoiuluslaeticollis* are more common in western Estonia or even restricted to this region characterised by milder maritime climate and calcareous soils e.g., *Geophiluscarpophagus*, *Boreoiulustenuis*, *Julusscanicus*, and *Cylindroiulusbritannicus*.

The currently known fauna of Estonian Diplopoda and Chilopoda is quite similar to the neighbouring regions. 79% of the species are shared with Finland and 87.5 % are shared with Latvia. The similarity to Latvian fauna may be in fact even higher, as several species occurring both in Estonia and Lithuania might be present also in Latvia. Estonian Symphyla and Pauropoda deserve further attention. At present, they remain too poorly known to allow for any comparisons.

## Supplementary materials

Supplementary material 1 Full collecting details for one finding from each locality. The locality numbers correspond to those in Table 1 and Figure 1. Data type: occurrence This is an open access article distributed under the terms of the Creative Commons Attribution License (CC BY 4.0), which permits unrestricted use, distribution, and reproduction in any medium, provided the original author and source are credited.Kaarel Sammet, Mari Ivask, Olavi Kurina
